# Adaptive model for rate of penetration prediction based on the dynamic correlation of influencing factors

**DOI:** 10.3389/fdata.2025.1676054

**Published:** 2026-01-05

**Authors:** Yonggang Deng, Xiaojing Zhou, Zixuan Feng, Xin Li, Hui Li

**Affiliations:** Research Institute of Safety, Environmental Protection and Quality Supervision and Inspection, Chuanqing Drilling Engineering Co., Ltd., Guanghan, China

**Keywords:** drilling parameter, correlation, dynamic evolution, rate of penetration prediction, adaptive model

## Abstract

**Introduction:**

Accurately predicting the rate of penetration (ROP) is a critical benchmark for evaluating operational efficiency in drilling operations, and it is necessary to optimize the drilling parameters and construct an accurate ROP prediction model. At present, the correlations between drilling operation parameters and the ROP are commonly evaluated using a static assessment, which overlooks dynamic changes in parameter correlations during drilling processes.

**Method:**

An adaptive ROP prediction model that incorporates depth-varying correlations of influential parameters is constructed. This model can automatically identify the dynamic correlations of the modeling parameters at different depths of well sections, and the optimal modeling parameters for adaptive training are selected based on the ranking of the correlation coefficients.

**Results:**

An analysis of 33 drilling parameters across 4,837 datasets collected from 4 wellbores in Sichuan. The comparison analysis revealed that at different well sections, the dynamic correlation coefficient of each parameter deviates significantly from the overall correlation coefficient. According to the proposed model, it can dynamically select key parameters and achieve self-update based on real-time data streams, avoiding the defect of traditional fixed-parameter models that ignore the dynamic changes of well sections.

**Discussion:**

Modeling comparison analysis revealed that in multiple rounds of prediction based on dynamic correlations, the prediction accuracy in 93% of the prediction rounds exceeded that of the overall correlation, indicating that the adaptive ROP prediction model with dynamic correlations has high application value.

## 1 Introduction

Drilling operations play a vital role in oil and gas exploration and development. The drilling process involves geological formations, drilling materials, equipment, and drilling tools, the control and optimization of the drilling process are very complex. Given the increasing depth of resource extraction, conventional drilling practices encounter significant challenges due to deep formations characterized by geothermal anomalies, high geostress, and complex geological structures, which are likely to lead to drilling accidents (Gan, 2019) and greatly affect drilling efficiency. In the evaluation and analysis of drilling efficiency, the prediction, control and optimization of the rate of penetration (ROP) are critical in improving drilling efficiency (Barbosa et al., 2019). However, the ROP should not be optimized to the maximum value. A reasonable ROP value is directly related to the smooth progress of a drilling project. Meanwhile, ROP is also the best choice for the estimation of rock mechanical parameters (Liu et al., 2021b). Some studies have noted that when the ROP exceeds the optimal range, it becomes more difficult to control the wellbore pressure and trajectory (Najjarpour et al., 2022).

Researchers at home and abroad have focused on ROP prediction and optimization. Currently, Currently, ROP models are commonly developed using three primary modeling approaches: theoretical models, statistical models, and machine learning models (Barbosa et al., 2019). The first category of theoretical models focuses on analyzing and modeling the entire drilling process. These models aim to identify and clarify the influence patterns of various parameters on the ROP. However, the main limitation of this type of model is that there are many factors affecting the ROP, and establishing an accurate physical model for description is difficult. To obtain more accurate analytical solutions, when establishing a theoretical model, it is usually necessary to introduce more assumptions to simplify the boundary conditions. These assumptions significantly affect the wide applicability of these models (Li et al., 2021). Statistical models focus on predicting the ROP from collected field data and generating a fitting curve equation. However, their primary limitation lies in numerous factors influencing the ROP, which vary across different drilling sites. Additionally, the non-linear impact of each parameter reduces the accuracy of ROP predictions. The development of machine learning models has benefited from the development of artificial intelligence technology in recent years. Through the introduction of different machine learning technologies, such as neural networks and support vector machines, and based on the large amount of data collected in the field, a multiparameter, non-linear model is established. By incorporating influencing factors into the ROP prediction models under linear trend conditions, the accuracy and efficiency of predictions significantly surpass those of conventional models. Consequently, some researchers have argued that machine learning models are a viable alternative for ROP prediction (Brenjkar and Biniaz Delijani, 2022). In addition, with the continuous increase in field data, several studies have noted that with increasing data, the accuracy of machine learning models is much higher than that of conventional statistical or theoretical models (Soares and Gray, 2019). Therefore, machine learning models have become widely used in real-world production scenarios in the current big data era. Zhang proposed an LSTM model combined with an attention mechanism, which highlights the parameter influence of key well sections through weight allocation (Zhang et al., 2022).

In the context of ROP prediction using machine learning techniques, correlation studies are often conducted in combination with correlation analysis and ROP prediction models, and the original data are generally preprocessed to improve the data reliability. Statistical methods are usually used in preprocessing to optimize a dataset, eliminate obvious dimensional errors, and improve accuracy (Tan et al., 2015). Li et al. analyzed 20,000 data points from 10 wells in China. By using the Pearson correlation coefficient method to evaluate ROP-related parameters, he divided the correlations into low, medium and high levels, providing a basis for drilling optimization (Li et al., 2021). Huang optimized a model by incorporating covariance results derived from correlation analysis and enhancing the correlation across multiple data types, leading to a 39% increase in the magnitude of the ROP model (Huang et al., 2021). Zhang proposed that the application of differential private feature selection in machine learning methods can reduce the influence of the internal correlation of parameters to a certain extent and improve data availability (Zhang et al., 2020). Zhao proposed a multivariate correlation analysis technique that incorporates the weighted key parameters, effectively reducing intrinsic data noise and improving data reliability (Zhao et al., 2022). Chen used the XGBoost model to screen the key parameters affecting ROP (such as drilling pressure and torque), excluded redundant parameters through feature importance ranking, and combined the particle swarm optimization algorithm to optimize drilling parameters (Chen et al., 2021). With respect to the ROP control process in other countries, machine learning methods have been used to regulate the parameters in the drilling process. To address the problems of incomplete and discontinuous data during the drilling process, Gan et al. applied the improved particle swarm algorithm and the improved bat optimization algorithm to establish an ROP prediction model based on a neural network and support vector machines, providing a new framework for ROP regulation under complex geological conditions (Gan et al., 2019a,b,c). Mohammed embedded rock mechanics equations as constraints into the data-driven model, which can ensure the rationality of predictions without requiring a large amount of labeled data (Mohammed et al., 2024).

In this study, on the basis of real-world data from four wellbores in a certain area of Sichuan, the concept of dynamic correlation was introduced, and calculations revealed that the overall correlation of relevant parameters significantly differed from the dynamic correlations at specific locations. Based on this finding, dynamic correlation was established as the core principle for selecting essential modeling parameters, replacing the conventional approach of fixed-parameter modeling with a dynamic modeling framework. Comparative analysis validated the advantages of this method.

## 2 Raw data pre-processing

### 2.1 Data sources

This study used a dataset comprising 4,837 entries with 33 parameter types collected from the production of 4 wells in a specific region of China. Although this number of data entries does not meet the requirements of conventional machine learning big data, statistics from relevant researchers show that the dataset size used by 51.7% of researchers in the field of ROP prediction modeling is in the range of [10^3^, 10^4^], and the median of this interval is 3,250 entries (Li et al., 2024). Accordingly, the amount of data used in this analysis is sufficient to achieve effective modeling results. The collected data can be divided into three types according to their type: inclinometer data, drilling engineering parameters and formation evaluation data. During the data integration process, measuring point depth and standard well depth were combined into a single parameter: well depth. Stratigraphic information was simplified to a stratigraphic number, with the subsection representing the smallest layer, and the corresponding number code was assigned as detailed in Table 1. As a result, the original data model was streamlined to 23 distinct data points, with the names and numbers of each parameter outlined in Table 2.

**Table 1:** Stratigraphic coding.

**No**.	**Code**	**Stratigraphic symbol**	**Eonothem**	**Erathem**	**Series**	**Formation**	**Member**	**Sub-member**	**Lithology**
1	100	J	Mesozoic	Jurassic	Lower	Ziliuwell	Zhenzhuchong	–	Mudstone (shale) and quartz fine sandstone
2	200	T3x	Mesozoic	Triassic	Upper	Xujiahe	–	–	Quartz sandstone
3	311	T2l	Mesozoic	Triassic	Middle	Leikoupo	Lei 1	Lei-1a	Dolomites
4	411	T1j	Mesozoic	Triassic	Lower	Jialingjiang	Jia-5	Jia-5b	Dolomite and salt-soluble breccia
5	412	T1j	Mesozoic	Triassic	Lower	Jialingjiang	Jia-5	Jia-5a	Dolomite and salt-soluble breccia
6	421	T1j	Mesozoic	Triassic	Lower	Jialingjiang	Jia-4	Jia-4d	Dolomite and salt-soluble breccia
7	422	T1j	Mesozoic	Triassic	Lower	Jialingjiang	Jia-4	Jia-4c	Dolomite and salt-soluble breccia
8	423	T1j	Mesozoic	Triassic	Lower	Jialingjiang	Jia-4	Jia-4b	Dolomite and salt-soluble breccia
9	424	T1j	Mesozoic	Triassic	Lower	Jialingjiang	Jia-4	Jia-4a	Dolomite and salt-soluble breccia
10	431	T1j	Mesozoic	Triassic	Lower	Jialingjiang	Jia-3	–	Thick limestone
11	441	T1j	Mesozoic	Triassic	Lower	Jialingjiang	Jia-2	Jia-2c	Dolomites
12	442	T1j	Mesozoic	Triassic	Lower	Jialingjiang	Jia-2	Jia-2b	Dolomites
13	443	T1j	Mesozoic	Triassic	Lower	Jialingjiang	Jia-2	Jia-2a	Dolomites
14	451	T1j	Mesozoic	Triassic	Lower	Jialingjiang	Jia-1	–	Limestone

**Table 2:** Parameter types collected in the field.

**Type**	**Name (parameter number)**
Recorded data while drilling	Drilling time (ROP, critical target parameter), standard well depth (1), large hook load (2), drilling pressure (3), torque (4), rotational speed (5), riser pressure (6), inlet flow (7), outlet flow (8), inlet temperature (9), outlet temperature (10), inlet conductivity (11), outlet conductivity (12), inlet density (13), outlet density (14)
Stratigraphic	Stratigraphic number (15)
Inclinometer data	Inclination angle (16), azimuth angle (17), true vertical depth (18), N coordinate (19), E coordinate (20), closed direction (21), closed distance (22), dogleg severity (23)

### 2.2 Raw data integration

**Figure 1:**
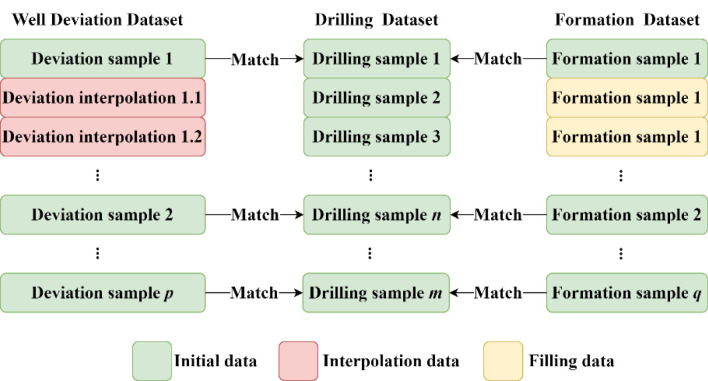
Schematic diagram of data stretching and interpolation.

The raw data collected for the study was not standardized, and there were obvious formatting errors. For example, data were collected while drilling at the standard interval of 1 m, whereas inclinometer data were collected at an interval between 0 and 30 m because of the use of different inclinometer equipment. The inclination accuracy was 13.45 m, the stratum information was a single piece of data per subinterval, and a complete wellbore had only 14 pieces of data at most. To facilitate data modeling and analysis, the above three types of data need to be integrated into a complete data matrix. Liu proposed the local linear kernel estimation method based on the exponentially weighted loss function, which is used to solve the problem of time asynchrony among multiple drilling parameters caused by different sampling frequencies of various sensors, so as to ensure data consistency (Liu et al., 2021a). Considering the demand for modeling data volume, in the present study, the drilling-time record was chosen as the reference framework, and the inclination data and formation data were stretched and reconstructed, respectively. Interpolation was performed to integrate the raw data (Figure 1).

**Figure 2:**
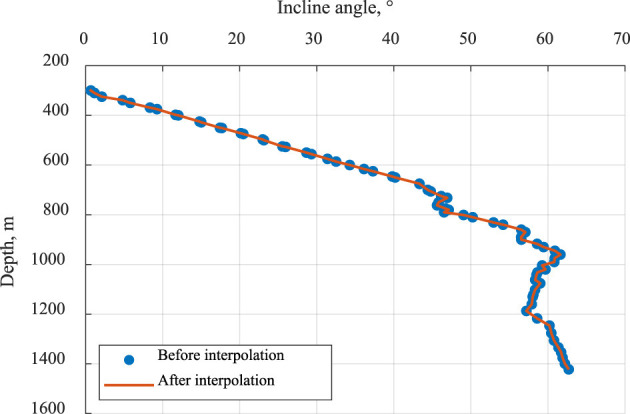
Comparison before and after interpolation using the inclination angle of Well No. 1 as an example.

The formation dataset was treated as a continuously distributed sequence across submembers, without abrupt structural discontinuities. Uniform values were inserted where data gaps existed. For deviation survey data, where drilling time entries occurred at the same depth, intersurvey intervals were aligned using Lagrange interpolation with corrections applied to the mean angle. Well inclination and azimuth angle were the two key variables interpolated using the Lagrange method, and the basic formula is shown in Equation 1. The inclination data from Well No. 1 were selected as an example, and the maps before and after interpolation were produced (Figure 2). This method provides a more accurate representation of the overall trend in data variation.


$$\begin{array}{l} y\left(x\right)=\overset{n}{\underset{k=1}{\sum }}y_{k}\left(\overset{n}{\underset{\begin{array}{c} j=1\\ j\neq k \end{array}}{\prod }}\frac{x-x_{j}}{x_{k}-x_{j}}\right) \end{array}$$ (1)

For other deviation survey data, the interpolation followed the regulations set by the Standardization Committee of China. The mean angle was used for correction. Let the depth of the specified position change be Δ*D*, the horizontal displacement be Δ*S*, and the N and E coordinates be; then, the basic calculation is shown in Equation 2.


$$\{\begin{array}{l} \Delta D=\left(1-\Delta \alpha^{2}/24\;\right)\Delta L\cos\alpha_{c}\\ \Delta S=\left(1-\Delta \alpha^{2}/24\;\right)\Delta L\sin\alpha_{c}\\ \Delta N=\left(1-\left(\Delta \alpha^{2}+\Delta \phi^{2}\right)/24\;\right)\Delta L\sin\alpha_{c}\cos\alpha_{c}\\ \Delta E=\left(1-\left(\Delta \alpha^{2}+\Delta \phi^{2}\right)/24\;\right)\Delta L\sin\alpha_{c}\sin\alpha_{c} \end{array}$$ (2)

## 3 Screening of critical factors based on the dynamic correlation

### 3.1 Overall correlation analysis

During the drilling process, both linear and non-linear relationships may exist between parameters and the ROP. Therefore, in the correlation analysis, four different correlation algorithms (as listed in Table 3) were used here. The target parameter is denoted *A*, and the drilling speed is *ROP*. The correlation between this parameter and the ROP can be calculated as *C*_*A*_, as shown in Equation 3.


$$\begin{array}{l} C_{A}=\\ \frac{|\text{co}{\text{r}}_{\text{P}}\left(A,ROP\right)|+|\text{co}{\text{r}}_{\text{S}}\left(A,ROP\right)|\text{+co}{\text{r}}_{M}\left(A,ROP\right)+\text{co}{\text{r}}_{C}\left(A,ROP\right)}{4} \end{array}$$ (3)

where cor_P_(*A, ROP*)/cor_S_(*A, ROP*)/cor_M_(*A, ROP*)/cor_C_(*A, ROP*) is the Pearson/Spearman/Mutual information/Chatterjee correlation coefficient for parameter A with the ROP.

**Table 3:** Selected correlation algorithms.

**Types**	**Calculation method**	**Remark**
Pearson	cor_*P*_ = cov(*X, Y*)/σ_*X*_σ_*Y*_	cov(*X, Y*) is the covariance for *X* and *Y*; σ_*X*_/σ_*Y*_ is standard deviation for *X* and *Y*
Spearman	$\text{co}{\text{r}}_{S}=1-\left(6\sum_{i=1}^{n}d_{i}^{2}\right)/n\left(n^{2}-1\right)$	*d*_*i*_is the difference in the rank value of the *i-*th data pair; *n* is the sample number;
Mutual information	cor_*M*_ = 2*MI*(*X, Y*)/(*H*(*X*)+*H*(*Y*))	*MI*(*X, Y*) is the mutual information for *X* and *Y*; *H*(*X*)/*H*(*Y*)is the entropy for X and Y
Chatterjee	$\text{co}{\text{r}}_{C}=\left(\sum_{i=1}^{n-1}|R\left(y\left(i+1\right)\right)-R\left(y\left(i\right)\right)|\right)/\left(n^{2}-1\right)$	*R*(*y*(*i*)) is the order of *Y* after sorting by *X*; *n* is the sample number;

For comparison and analysis, first, the overall correlation between each parameter in the current dataset and the ROP was calculated, as shown in Figure 3. Based on this evaluation (Li et al., 2021), nine parameters exhibited strong correlations (correlation coefficient >0.6). These parameters, ranked from strongest to weakest are as follows: torque, rotation speed, azimuth angle, outlet temperature, closure azimuth, dogleg severity, E coordinate, inlet temperature and inclination angle. Other parameters demonstrated moderate correlations (correlation coefficient of 0.3–0.6), indicating less pronounced but still relevant relationships.

**Figure 3:**
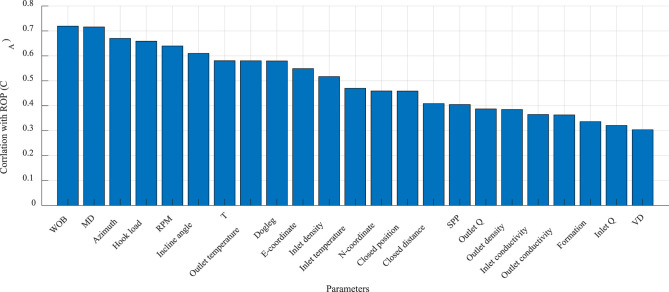
Overall correlation analysis of the current dataset.

### 3.2 Dynamic correlation analysis

**Figure 4:**
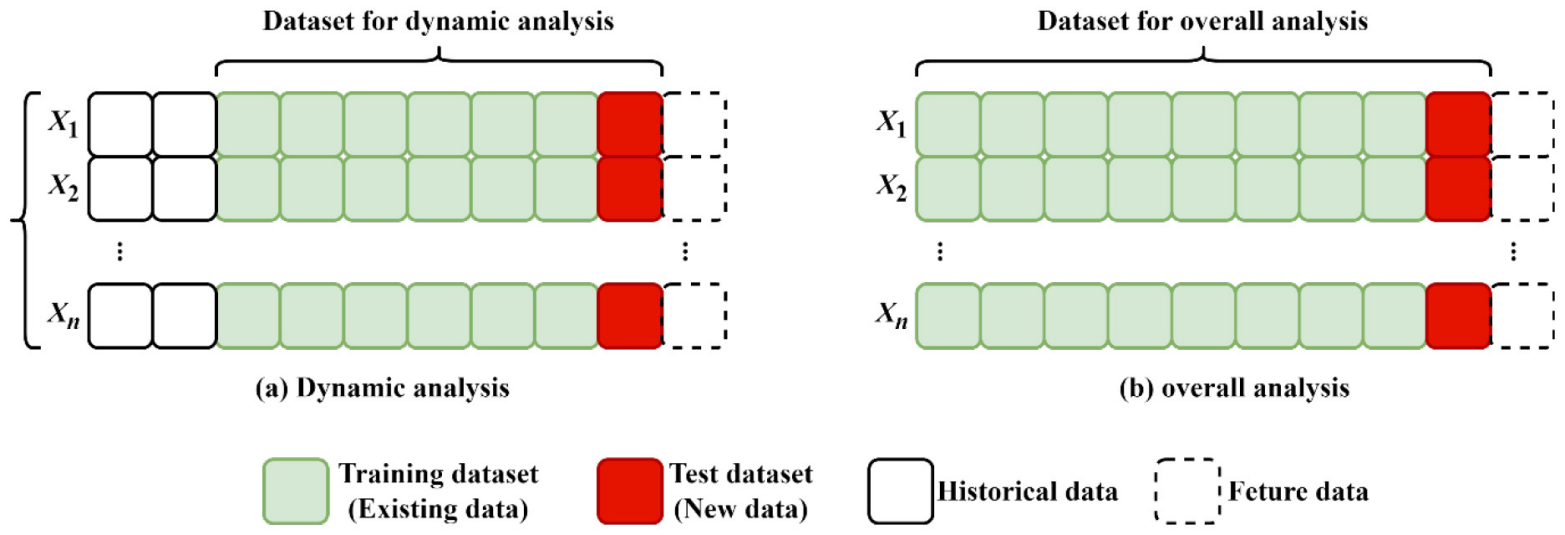
Comparison of the principles of dynamic correlation analysis **(Left)** and overall analysis **(Right)**.

With the introduction of real-time data streams during the drilling process, modeling and analysis datasets are constantly changing. Thus, overall analysis methods must also change constantly. However, as the amount of data increases, the previous data may have an unknown influence on the current data, reduces modeling accuracy. This study introduces the use of dynamic correlation analysis to remove the influence of previous data. The basic principle of dynamic correlation analysis is shown in Figure 4. First, the size of the dynamic analysis dataset is defined. As real-time data are continuously incorporated, earlier data, particularly those furthest from the latest entries, are gradually removed from the data. This approach ensures that the model and analysis remain responsive and sensitive to the most current data in real time.

Building on the dynamic analysis principle (Figure 4), the size of the dynamic analysis dataset has a significant impact on the outcomes of the analysis. Since the primary aim of the dynamic correlation analysis in this study is to establish an ROP prediction equation, the size of the dataset capable of supporting accurate ROP prediction is used to define the dynamic analysis dataset. According to relevant surveys, at present, 82.7% of researchers divide ROP prediction datasets into three sizes: 1,000–10,000, 100–1,000 and < 100, representing 51.7%, 20.7% and 10.3% of the total, respectively. The median sizes of these respective datasets are 3,250, 315 and 78 entries, respectively (Li et al., 2024). Considering that this study uses 4,837 records for 4 wells, the median of smallest group, 78 records, was selected as the size of the dynamic analysis dataset.

**Figure 5:**
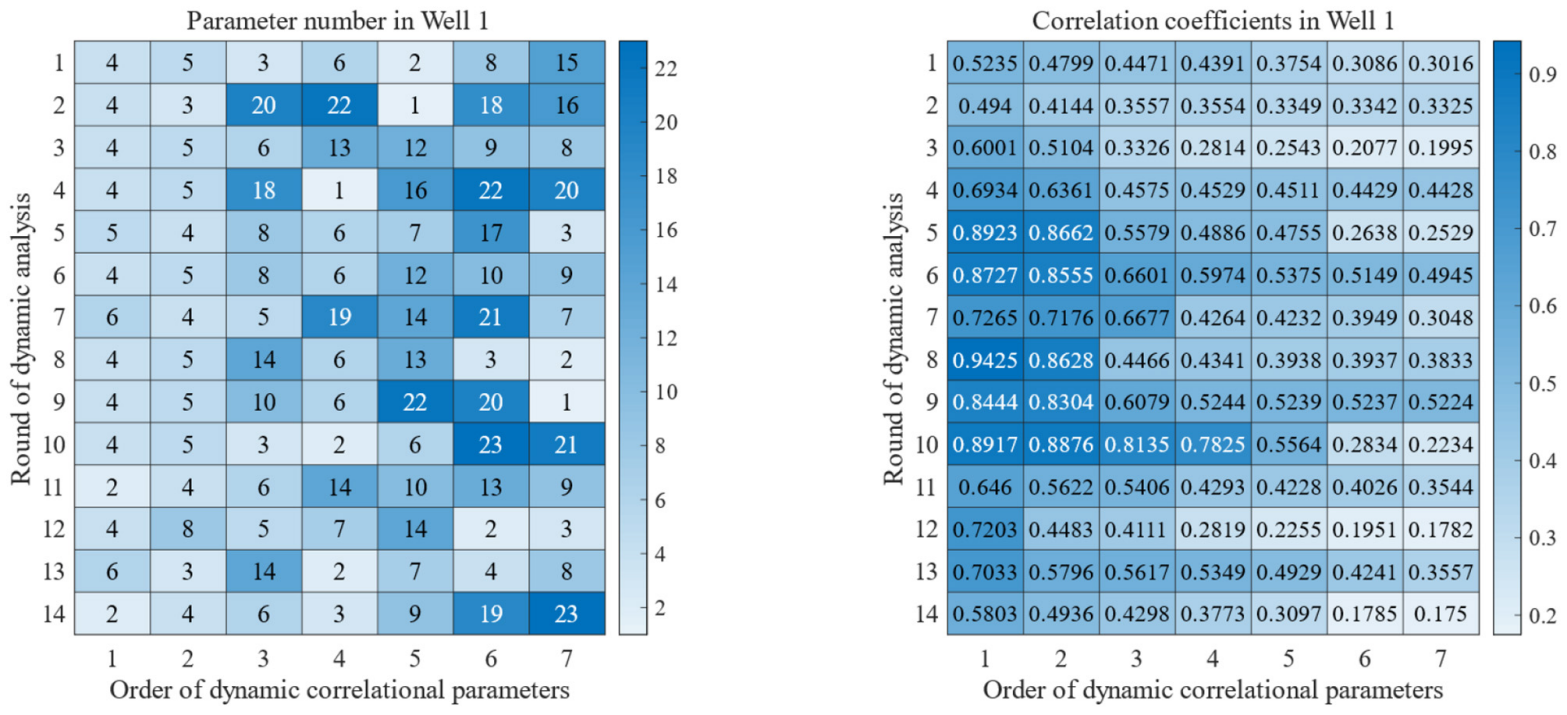
Dynamic correlation optimization parameters **(Left)** and corresponding correlation coefficients **(Right)** for Well No. 1.

Relevant survey findings indicate that most ROP prediction models involve 6–10 modeling parameters, with a median of 7 parameters (Li et al., 2024). Therefore, in the present study, we analyze only the first seven parameters based on the correlation ranking. Figure 5 present the results for Well No. 1. In this Figure, the horizontal axis of each Figure represents the top seven parameters ranked by correlation for each analysis, and the vertical axis represents different analysis rounds along the well depth direction. Across different analysis rounds, the first seven parameters with highest correlation rankings, especially the 3rd to 7th parameters, change significantly. From a correlation perspective, the correlation parameters in different rounds change significantly; for parameters at the same position, the correlation values fluctuate by approximately 0.3 in different rounds of analysis.

**Figure 6:**
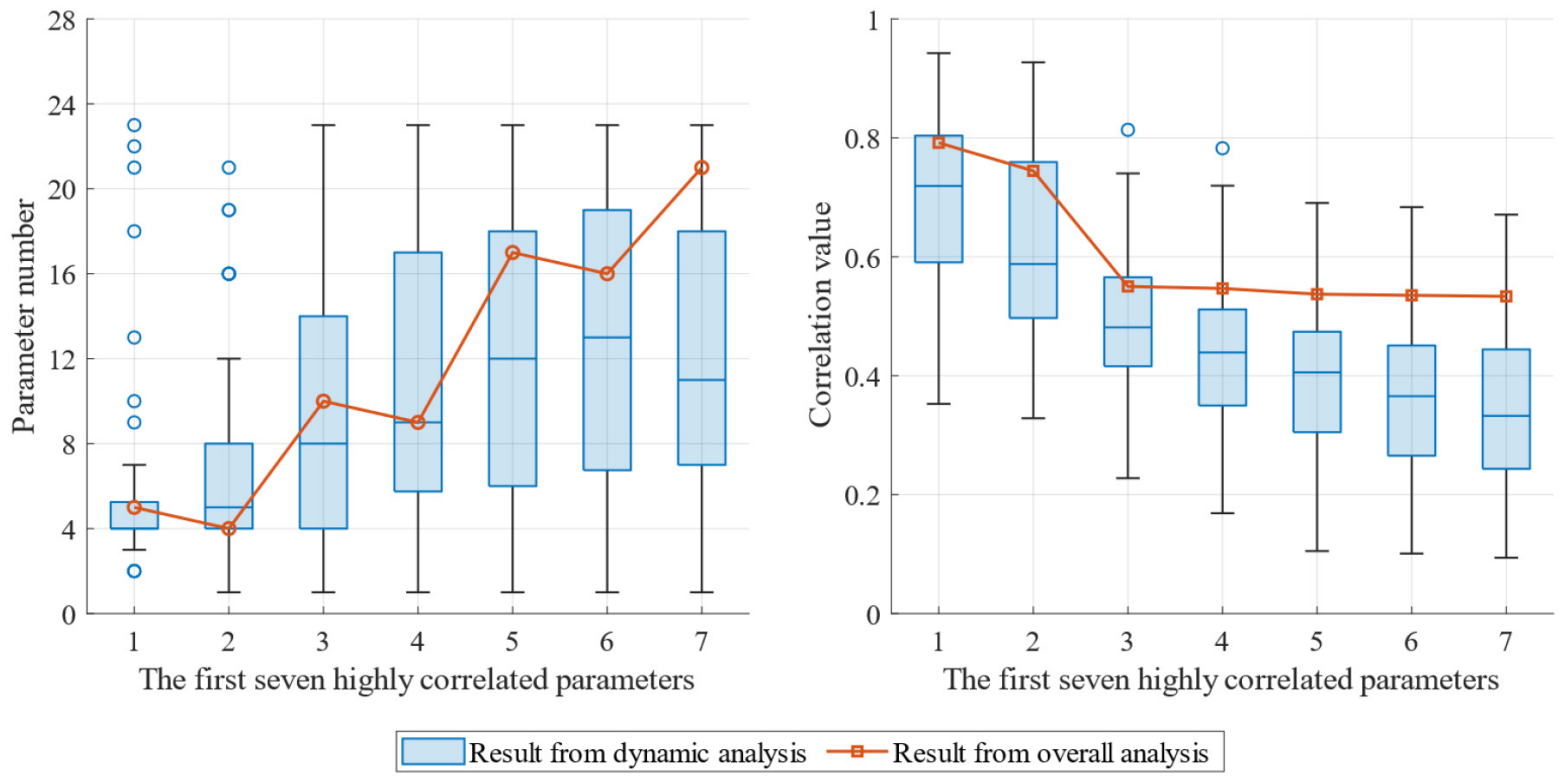
Distribution of dynamic correlation optimization parameters **(Left)** and respective correlation coefficients **(Right)** in different rounds.

Twelve to fourteen rounds of dynamic correlation analysis were performed for each of the 4 wells. The distributions of the first seven relevant parameters across all rounds and their respective correlation coefficients are listed in the box plot shown in the Figure 6. From the distribution of relevant parameters, as the correlation decreased (from the first to the seventh), the types of relevant parameters varied more extensively and, the distribution becomes wider (Figure 6 left). From the perspective of the correlation coefficient, the first seven parameters identified through the overall correlation analysis can be divided into two groups. In the first group, the first- and second-best parameters had correlations of 0.79 and 0.74, respectively; in the second group, the remaining five parameters were evenly distributed, with correlation coefficients ranging between 0.53 and 0.55. For the dynamic correlation analysis, there were significant differences in the results across various wellbores, different evaluation rounds, and the overall correlation. The medians of the correlation coefficients of the top 2 influencing factors were concentrated at approximately 0.72 and 0.58, but in some rounds, the highest correlations reached 0.94 and 0.93, respectively, whereas the correlation distributions of the latter five relevant parameters were significantly lower than that from the overall analysis.

## 4 Dynamic correlation-based adaptive ROP prediction model

### 4.1 Adaptive model architecture process

**Figure 7:**
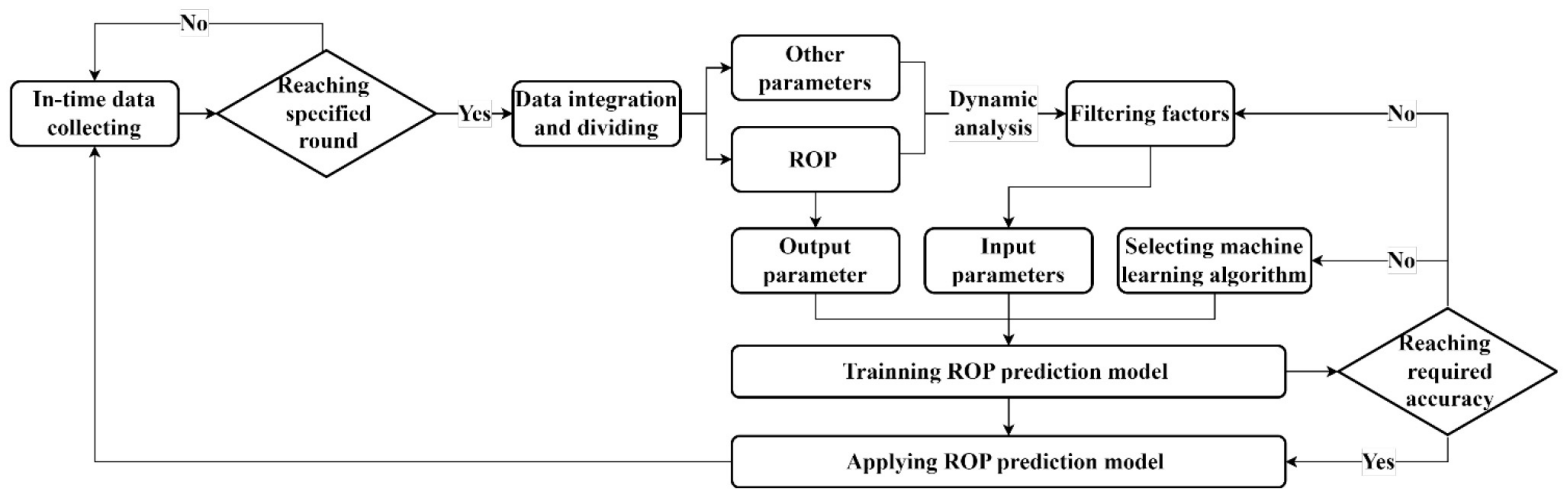
Architecture principle of the dynamic correlation-based ROP prediction model.

The principle of adaptive model architecture is shown in Figure 7. Based on the acquisition process of the real-time data stream, the core steps of the model are outlined as follows:

① Determine whether the current real-time data collection has reached the minimum number of entries to initiate dynamic correlation analysis. In this study, the size of the dataset is limited to 78 entries owing to data limitations. However, the size of the dataset can be increased depending on the size of the real-time data stream in the actual process. If there is a sufficient amount of data, the data collection process continues. When the size of the dataset meets the standard, the modeling process begins;

② The first step in the modeling process is data integration. During this step, data with different acquisition intervals and precisions are integrated into a unified analysis matrix. After the integration, the ROP data are separated from other data for future use.

③ After the data are integrated, the correlation between each data point and the ROP is computed. The parameters are then ranked according to their correlation strength.

④ After the data are screened, the selected influencing factors are used as input parameters, and the ROP is used as the output parameter. A selected machine learning algorithm is applied for training. After training, if the model accuracy does not meet the standard, the influencing factors are rescreened and the model is retrained or a more appropriate machine learning algorithm is selected. Once the accuracy of the model meets the standard, the model is used for prediction, and the data collection process begins for the next round of analysis.

### 4.2 Comparison of modeling effects of the adaptive ROP prediction model

On the basis of the test data, the algorithm of multilayer perceptron (MLP) was chosen to construct and analyze the adaptive ROP prediction model. The main reason for choosing the MLP algorithm is its widespread applicability. According to relevant research, 52.4% of studies using machine learning to predict drilling rate over the past decade have used artificial neural network (ANN) algorithms, and 61.1% of these applications have employed the MLP algorithm (Gan et al., 2019c). Therefore, using the MLP algorithm to verify dynamic correlations demonstrates the adaptability of this study to a significant extent.

**Figure 8:**
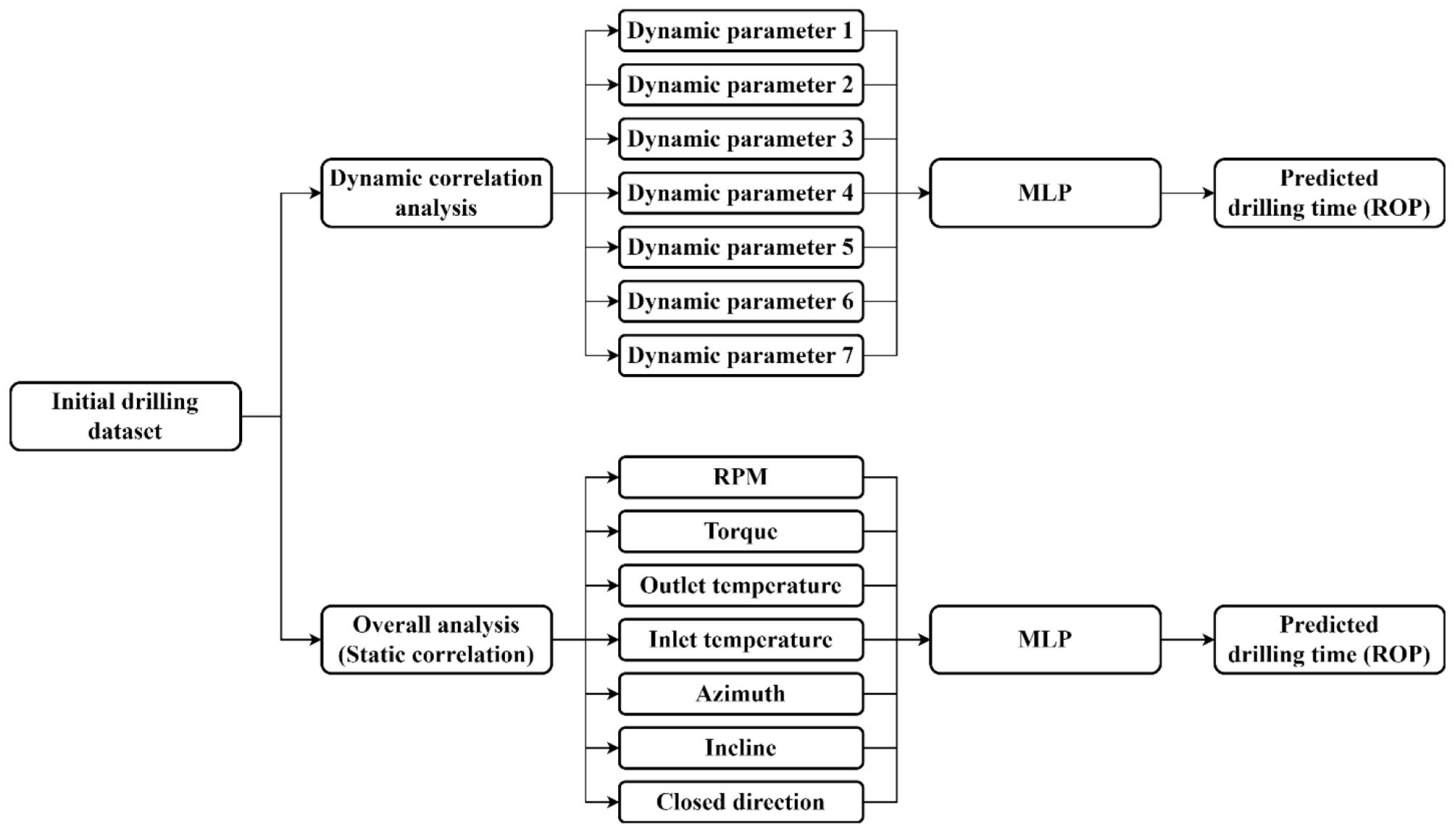
Construction of the dynamic correlation ROP prediction model and overall correlation ROP prediction model.

In this dynamic modeling approach, different correlation parameters were used across different rounds for different wells (Figure 8 left). In contrast, in the overall modeling approach, the top seven parameters identified from the overall correlation analysis are selected as input parameters (Figure 8, right). To evaluate the performance of the prediction model, the coefficient of determination (*R*^2^) was applied, shown in Equation 4. This metric measures the degree of fit between the predicted and actual values. The *R*^2^ value ranges between 0 and 1, and the closer to 1 it is, the better the degree of fit of the regression model to the observed values.


$$\begin{array}{l} R^{2}=1-\overset{n}{\underset{i=1}{\sum }}{\left(y_{i}-\overset{\wedge }{y_{i}}\right)}^{2}/\overset{n}{\underset{i=1}{\sum }}{\left(y_{i}-\bar{y_{i}}\right)}^{2} \end{array}$$ (4)

**Figure 9:**
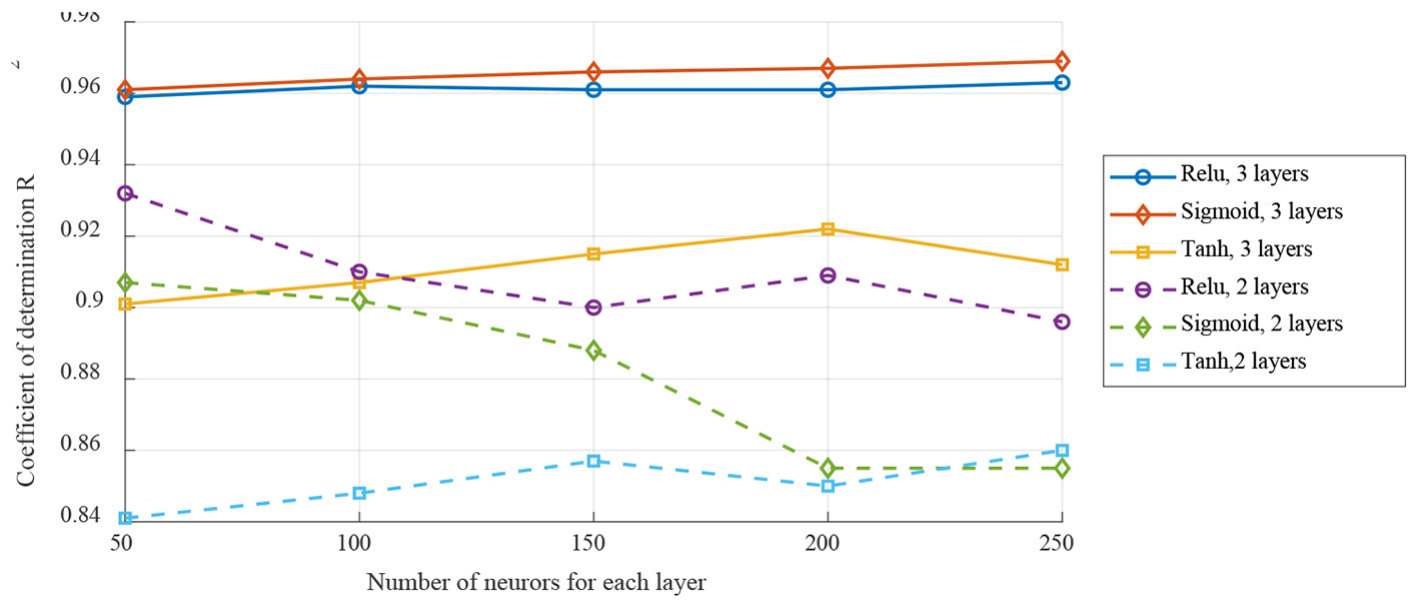
Selection of MLP structure parameters.

To make a better comparison, using the overall analysis to select the MLP structure parameters, which the dynamic analysis using the same parameters. Three structure parameters for MLP were selected here including the activation function, the number of network layers and the number of neurons for each layer. As shown in Figure 9, the testing result indicated that using the activation function of Sigmoid, 3 hidden layers and 250 neurons for each layer will achieve the best performance.

For the modeling data grouping, the predictive modeling utilized a basic 7:3 static grouping approach, where 70% (3,386 entries) of all original data were randomly selected as the training dataset, and 30% (1,451 entries) were used as the test dataset. For dynamic analysis and modeling, 78 data points were used in each analysis; therefore, 55 data points were used for training, and 23 data points were randomly selected.

**Figure 10:**
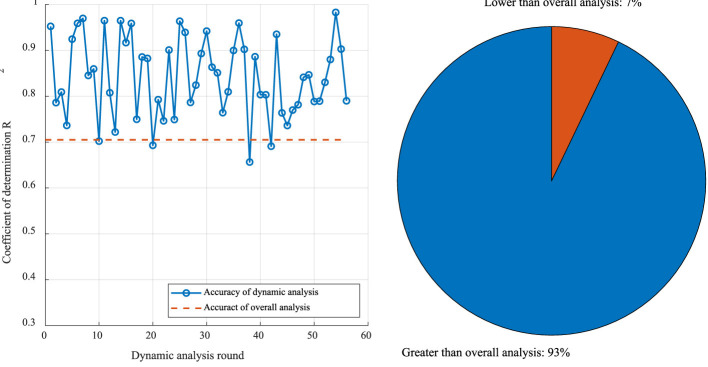
Comparison of the prediction accuracies of dynamic correlation modeling and the overall analysis.

**Figure 11:**
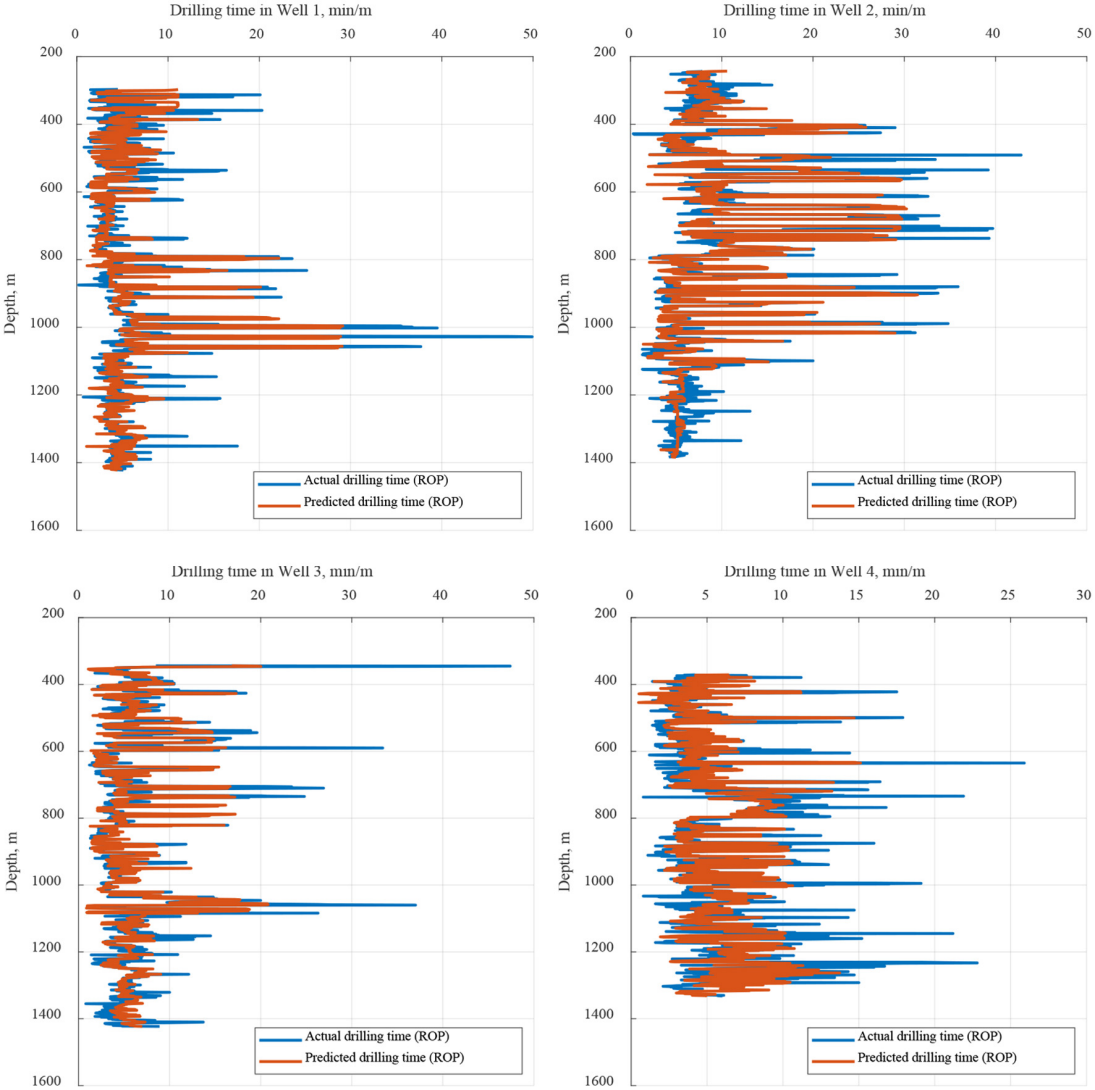
Comparison of the prediction results for Wells 1–4. **(Upper left)** Well 1; **(Upper right)** Well 2; **(Lower left)** Well 3; and **(Lower right)** Well 4.

The modeling analysis results are shown in Figure 10. The prediction accuracy of the fixed parameters, based on the overall correlation analysis, was 0.71. This was followed by the prediction accuracy of the dynamic correlation and the overall correlation in the base comparison. In different rounds of dynamic correlation prediction, the accuracy of 93% achieved by the dynamic analysis model was higher than that for the overall analysis, and the prediction accuracy of 65% (36 times) exceeded 0.8, meeting the standard required for daily application. The final dynamic analysis model was used to predict the ROP for the four wells selected in this study. The predicted data were compared with the actual data (Figure 11). The prediction model established on the basis of the dynamic correlation analysis in this study generated data with a high degree of agreement with the actual data.

### 4.3 Performance comparison of different ML algorithms with dynamic correlation analysis

To further validate the universality and effectiveness of the dynamic correlation analysis in ROP prediction, this study compared the prediction accuracy of several mainstream machine learning algorithms under the same dynamic modeling framework. The selected algorithms included Random Forest (RF), eXtreme Gradient Boosting (XGBoost), and Long Short-Term Memory (LSTM) networks, and were compared against the Multilayer Perceptron (MLP) used in the original analysis. All models adopted the same dynamic correlation parameter screening mechanism, i.e., selecting the top 7 most relevant parameters in each analysis window as input features. The specific configurations for each algorithm were as follows: Random Forest was set with 100 trees and a maximum depth of 10; XGBoost used a learning rate of 0.1, a maximum depth of 6, and 100 trees; the LSTM model consisted of a single layer with 50 hidden units, followed by a fully connected layer and utilized the Adam optimizer. All models were trained and evaluated using the same 70:30 training-testing split within each dynamic analysis window.

The prediction accuracy, evaluated using the coefficient of determination (*R*^2^), is summarized in Table 4. Among the compared algorithms, XGBoost demonstrated the best performance, achieving the highest average *R*^2^ (0.87) and the highest proportion of prediction rounds (72%) where accuracy exceeded 0.8, highlighting its strong capability for feature selection and non-linear fitting. MLP also showed competitive results, closely following XGBoost, which further validates the robustness of the dynamic correlation framework. Random Forest provided stable and reliable performance, particularly advantageous in scenarios with limited data. While LSTM possesses inherent strengths for temporal modeling, its performance in this specific dynamic window analysis was slightly lower, potentially due to uneven data sampling intervals or less pronounced long-term dependencies in the dataset. In conclusion, the dynamic correlation analysis method significantly enhances the prediction accuracy not only for MLP but also for other powerful algorithms like XGBoost and Random Forest, demonstrating strong adaptability and substantial practical value for drilling optimization.

**Table 4:** Performance comparison of different machine learning algorithms.

**Algorithm**	**Average *R*^2^**	**Maximum *R*^2^**	**Percentage of rounds with *R*^2^ > 0.8**
Random forest	0.83	0.92	58%
XGBoost	0.87	0.98	72%
LSTM	0.80	0.87	54%
MLP	0.84	0.97	65%

## 5 Discussion

### 5.1 Key findings

Our analysis indicates that the overall correlation coefficients between drilling parameters and ROP, commonly used in traditional models, do not adequately capture the localized and depth-variant effects of these parameters. For example, while parameters such as torque, rotational speed, and azimuth exhibit strong overall correlations with ROP, their impacts vary significantly across depth intervals. This variability highlights the need for dynamic modeling approaches that can adapt to changing downhole conditions.

Dynamic correlation analysis further revealed that the most significant influencing parameters identified in each analysis cycle varied not only in type but also in the magnitude of their correlations with ROP. This suggests that fixed-parameter models may overlook key parameters that are only important under specific geological or operational conditions. The adaptive model's ability to selectively incorporate these parameters in real time enhances its superior predictive performance, as confirmed by the comparative analysis of multiple ML algorithms in Section 4.3.

The adaptive ROP prediction model proposed in this study has important practical implications for drilling optimization. By accurately predicting ROP with different parameters in different depths, the model can help drillers adjust operational parameters in real time, maximizing efficiency while minimizing risks such as tool wear, wellbore instability, and non-productive time. The model also performs well with relatively small datasets (78 data points per analysis), making it suitable for oilfields with limited historical data.

### 5.2 Limitations and future work

While this study achieved encouraging results, several limitations remain. First, the model was trained and tested based on data from only four wells in a single geological region. Its generalizability to other formations or drilling environments remains to be verified. Second, the dynamic analysis window size (78 data points) was selected based on the survey median; however, the optimal window size may vary depending on data quality and drilling complexity. Third, the performance of LSTM might be further explored with optimized hyperparameters or different architectures more suited to the specific data characteristics.

Future work should focus on validating the model in diverse geological settings and incorporating more advanced machine learning algorithms, such as recurrent neural networks (RNNs) or Transformers, which are better suited for processing time series data. Furthermore, combining physical models with data-driven approaches could further enhance the model's interpretability and robustness.

## 6 Conclusions

Through dynamic correlation modeling analysis of four wells from the Sichuan field, this study yields the following key conclusions:

(1) Based on the integration of field data and the overall correlation analysis of all the data, the data from the four wells collected in this study generally exhibited medium to high correlations. Among the 33 parameters related to the ROP (correlation coefficient > 0.6), 9 parameters demonstrated strong linear relationships with the ROP. The remaining parameters were all moderately correlated parameters, with correlation coefficients between 0.3 and 0.6.

(2) Based on the dynamic correlation analysis, the dynamic correlation and overall correlation of each parameter significantly differed across different well sections. Using the statistical median of 78 data points from the ROP prediction modeling dataset as the dynamic analysis scale, the top seven parameters ranked by correlation were compared in each round of analysis. Over time, numerical fluctuations in both parameter type and correlation coefficient became increasingly pronounced.

(3) The first seven parameters were screened via overall correlation and dynamic correlation for modeling analysis. The modeling prediction accuracy based on dynamic correlation was significantly higher than that of the ROP prediction model established by overall correlation. The prediction accuracy in the dynamic correlation modeling rounds was higher than that in the overall correlation modeling prediction.

(4) Experimental results indicate that even when modeling with only 78 data points, its accuracy is still higher than that of the overall correlation model based on 4,837 data points, and this characteristic makes it particularly suitable for drilling scenarios with limited historical data.

## Data Availability

The original contributions presented in the study are included in the article/supplementary material, further inquiries can be directed to the corresponding author.
